# Can Kinect aid motor learning in sportsmen? A study for three standing techniques in judo

**DOI:** 10.1371/journal.pone.0210260

**Published:** 2019-02-06

**Authors:** Cezary Sielużycki, Jarosław Maśliński, Patryk Kaczmarczyk, Rafał Kubacki, Wojciech B. Cieśliński, Kazimierz Witkowski

**Affiliations:** 1 Department of Biomedical Engineering, Faculty of Fundamental Problems of Technology, Wrocław University of Science and Technology, Wrocław, Poland; 2 Faculty of Computer Science and Management, Wrocław University of Science and Technology, Wrocław, Poland; 3 Faculty of Sport Sciences, University School of Physical Education in Wrocław, Wrocław, Poland; Hochschule Trier, GERMANY

## Abstract

Our objective was to examine how exercises with the second generation of the Microsoft Kinect sensor may aid in the process of motor learning in young judo practitioners. We addressed improvements in spatio-temporal accuracy during execution of three standing techniques in judo, in a simple paradigm designed to study short-term practice effects. Two groups of judokas, 12 athletes each—one aided with Kinect and our dedicated software *vs* a group of controls—were asked to mimic previously recorded master-level performances of the three techniques, established as benchmarks by a two times world champion in judo. In five training sessions, athletes of the aided group used a virtual-reality setup in which they trained with a virtual representation of the master displayed on a large screen with a simultaneous real-time visualisation of their own movements in the form of an avatar based on body joint localisation, as determined by Kinect, which also measured their performance. The control group used Kinect in the 1st and 5th session, which was necessary for the measurements that constituted the basis for subsequent statistical comparisons, whereas the 2nd, 3rd, and 4th session in this group was guided by a coach, without the use of the Kinect setup. In addition, athletes of the two groups had unrestricted access to a video recording of the master performing the three throws. We found statistically significant improvements (*p* < 0.05) in the accuracy of executing the three techniques between the 1st and the 5th training session for the aided group but not for the control group. We conclude that incorporating Kinect based exercises into a judo training programme may be a useful means to supporting motor learning, therefore enhancing training efficiency, and thus improving performance.

## Introduction

The Kinect sensor by Microsoft, initially intended for enhancing user experience in video games, is gaining increasing interest from the scientific community, primarily in disciplines related to biomechanics. Since it is capable of real-time 3D spatial localisation of human body joints in a 3D scene, it represents a powerful tool in a range of experimental paradigms aimed at studying various aspects of human body kinematics. The low cost of Kinect makes it a very interesting, commonly available alternative to expensive, and hence often unavailable, systems for motion tracking. This availability of Kinect was the main motivation of our study.

A direct comparison of the accuracy of Kinect against marker-based optical motion capture systems is problematic [1]. This is because of the different origins of errors stemming from the different operating principles. Namely, Kinect generates volumetric silhouettes which are parsed and reduced to simplified skeletons in a fairly complex computational routine. Marker-based systems, on the other hand, even if theoretically accurate, suffer from errors caused by surface movement artefacts (markers are taped to the skin, which moves with respect to the skeleton; the marker adhesive can also loosen) and human error in marker placement [1]. Kinect avoids such errors as well as the time and need for expert operators to place makers accurately and precisely. A diligent, technically oriented discussion on the comparison of Kinect to a gold-standard marker-based Qualisys system can be found in [1]. Another comparison, against a gold-standard Vicon system [2], concluded that Kinect has the potential to be used as a reliable and valid clinical measurement tool (see, e.g., Table 2 in [2]). Moreover, Kinect requires very limited set-up time, whereas marker-based capture systems require about half an hour for placement and calibration [1]. These benefits can translate into a sound motivation to use Kinect in movement studies.

Indeed, the device has been applied to the analysis of gait differences between children and adults [3] or between healthy individuals and Parkinson’s disease patients [4]. Given these capabilities, a natural application appears to be rehabilitation of patients after injuries or strokes [5]. Kinect has also been used to study motor control in ageing [6, 7], body balance [7], dance [8], and to assess object control skills [9].

In this paper we address the question as to whether Kinect can be an efficient aid in professional sports, focusing on the example of judo. Judo training involves the rehearsal and effective execution of given techniques in simulated combat, i.e. without the resistance of an opponent. Simulated execution is intended to support the development of motor structures, on the level of both the nervous system and muscles, which is hoped to result in improving the performance of the practitioner in a real fight. Such simulations can potentially be supported by computerised technologies in that the improvement, understood as the minimisation of errors, in learning (mastering) advanced bodily techniques can be achieved by optimising sensorimotor transformations through an optimised interplay of feedforward and feedback control mechanisms [10–12], thus improving performance. The key mechanism here is observational learning [13], in which individuals copy an action, e.g., when they match their movements to those of others [13]. In our experiment, they were trying to match to the virtual representation of the previously recorded reference movements of a judo master. The critical advantage of using the system presented in our work is the real-time visual feedback, as well as the post-hoc feedback, both of them enabling the athletes to match their joints to the joints of the master during the process of copying an action, namely, a judo throw.

Inaccuracies in movement execution stem from variability in the sensory inputs and motor outputs or from errors in the internal representations of this information [11]. When learning a completely new, demanding technique, even a master-level athlete encounters difficulties. Therefore, in a series of trials, only one realisation might be close to perfect, and yet, likely only accidentally. However, this semi-perfect realisation, as assessed by, for example, an experienced coach, can be selected to serve the role of the reference in further training, especially in the absence of another reference pattern. Even though the initial attempts to follow the reference performance will be characterised by large spatio-temporal variance, one may expect that this variance will decrease steadily with practice and hence a quasi-deterministic performance at the reference level will eventually be achieved.

Motivated by the low cost and hence broad availability of the Kinect sensor, we explore its potential in supporting motor performance and motor learning [14, 15] in young judo athletes. We focus on three selected standing techniques of the throwing techniques in judo [16, 17]. We analyse athletes’ improvements in spatio-temporal accuracy of body movements [18] in a series of training sessions aided by Kinect. During these sessions, athletes were asked to follow reference performances of judo techniques based upon recordings acquired with a recognised judo master (twice world champion). In our discussion, we point out practical limitations of using Kinect and also potential constraints as far as the transference of the enhanced skills to the real world is concerned. We also sketch future directions.

To the best of our knowledge, this is the first study incorporating Microsoft Kinect as a tool to aid training efficiency in judo in a motor learning paradigm aimed at improving performance.

## Methods

### Participants

Two groups, each consisting of 12 young healthy male athletes, participated in the study, which had been approved by the Senate Research Ethics Committee at the University School of Physical Education in Wrocław. Written informed consent was obtained from participants of the study and from parents for adolescent participants. One of the two groups, named *Aided* (age range 12–28 y/o, mean 19 y/o, standard deviation 4 yrs; judo-experience range 4–15 yrs, mean 10 yrs, standard deviation 3 yrs), was aided by Kinect in five training sessions. The other group, named *Controls* (age range 13–27 y/o, mean 18 y/o, standard deviation 4 yrs; judo-experience range 4–18 yrs, mean 10 yrs, standard deviation 4 yrs), consisted of other athletes, who also participated in five training sessions but those used Kinect only in the 1st and 5th session, with the three in-between sessions being guided by a coach. Involving Kinect in the 1st and 5th session in the control group was necessary for the measurements that constituted the basis for subsequent statistical comparisons of the performance of the two groups of athletes (see Results).

### Procedure

**Fig 1:**
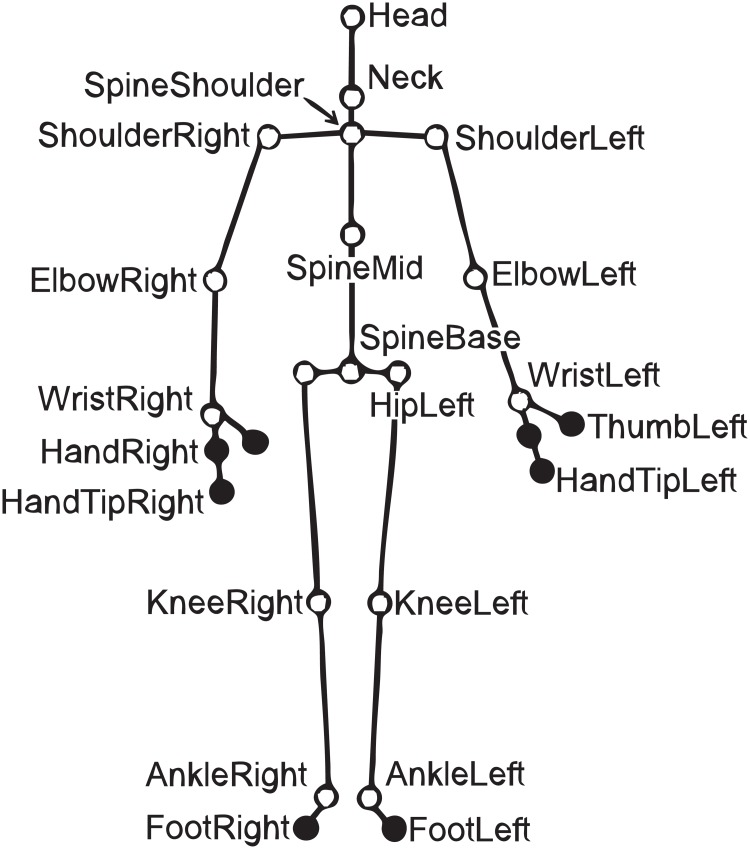
Twenty five joints of the virtual skeleton characteristic to the second generation of Kinect. The 17 joints that were analysed in this study (see Methods) are marked as white, as opposed to the skipped eight joints, which are marked as black.

We used the 2nd-generation Kinect sensor and our own purposely written software in C#, named InterAction, which makes use of the SDK libraries by Microsoft. Kinect can recognise and parametrise a human body in terms of 3D locations of 25 joints of a virtual skeleton (see Fig 1) that is assigned to the body of a person who is assumed to be facing the Kinect. Measurements are embedded in the Cartesian coordinate system whose origin is located in the centre of the imaging plane defined by the imaging sensor behind Kinect’s lens. Hence, the *x*- and *y*-directions reflect the horizontal and, respectively, vertical span of the scene, while the *z*-axis, which is parallel to the optical axis of the lens, reflects the depth, i.e. the distance of the parametrised body joints from Kinect.

In addition, during all sessions, all athletes in the two groups had unrestricted on demand access to a video recording of the master performing the three throws, which had been recorded simultaneously by Kinect during the parametrisation of the master. They could watch it in either slow motion or at normal speed, depending on their preferences. We assumed we should give the athletes full freedom in deciding how many times and how long they could watch the video recording of the master. This strategy was motivated by the fact that each of the two groups consisted of athletes of different age and judo experience and hence it appeared plausible to let them decide freely how much support of this kind they would need.

Each session lasted about 20 mins altogether, which stemmed mainly from the number of throw realizations to perform. This time was very similar for each athlete, irrespective of the group.

Since Kinect cannot see those joints that are located in optical shade, and hence infers rather than traces their locations in space, we focused on three standing techniques that are relatively unaffected by this problem in order to obtain reliable measurements. These three techniques, referred to as throws throughout the paper, were: 1) *de-ashi-barai* (known also *as deashi-harai*), 2) *osoto-gari*, and 3) *ouchi-gari* (all names in romanised Japanese), belonging to the group of foot and leg techniques (*ashi-waza*), although they naturally engage the entire body [16–18]. An athlete had to perform each of the three throws ten times within a session (not including three warm-up trials at the beginning), resulting in a total of 30 trials per session, altogether 150 trials across all five sessions. For each throw, athletes’ performance was evaluated with respect to the previously recorded performance of a two times world men’s champion in judo, referred to as the master from now on. During data acquisition by the software, his body was parametrised in real-time (30 frames per second) in terms of spatial 3D locations of the 25 body joints.

A justified potential question would be whether the acquisition speed of 30 frames per second is sufficient for capturing a judo throw. With this in mind, we asked the master to perform the reference realizations of the throws accordingly. Namely, the reference performances were not particularly fast, although not particularly slow either. The speed was typical for a normal, traditional training focused on accuracy rather than speed, because in the considered paradigm, emphasis was put on accuracy. A single realization lasted about a second, which translates to a reasonable number of about 30 frames.

**Fig 2:**
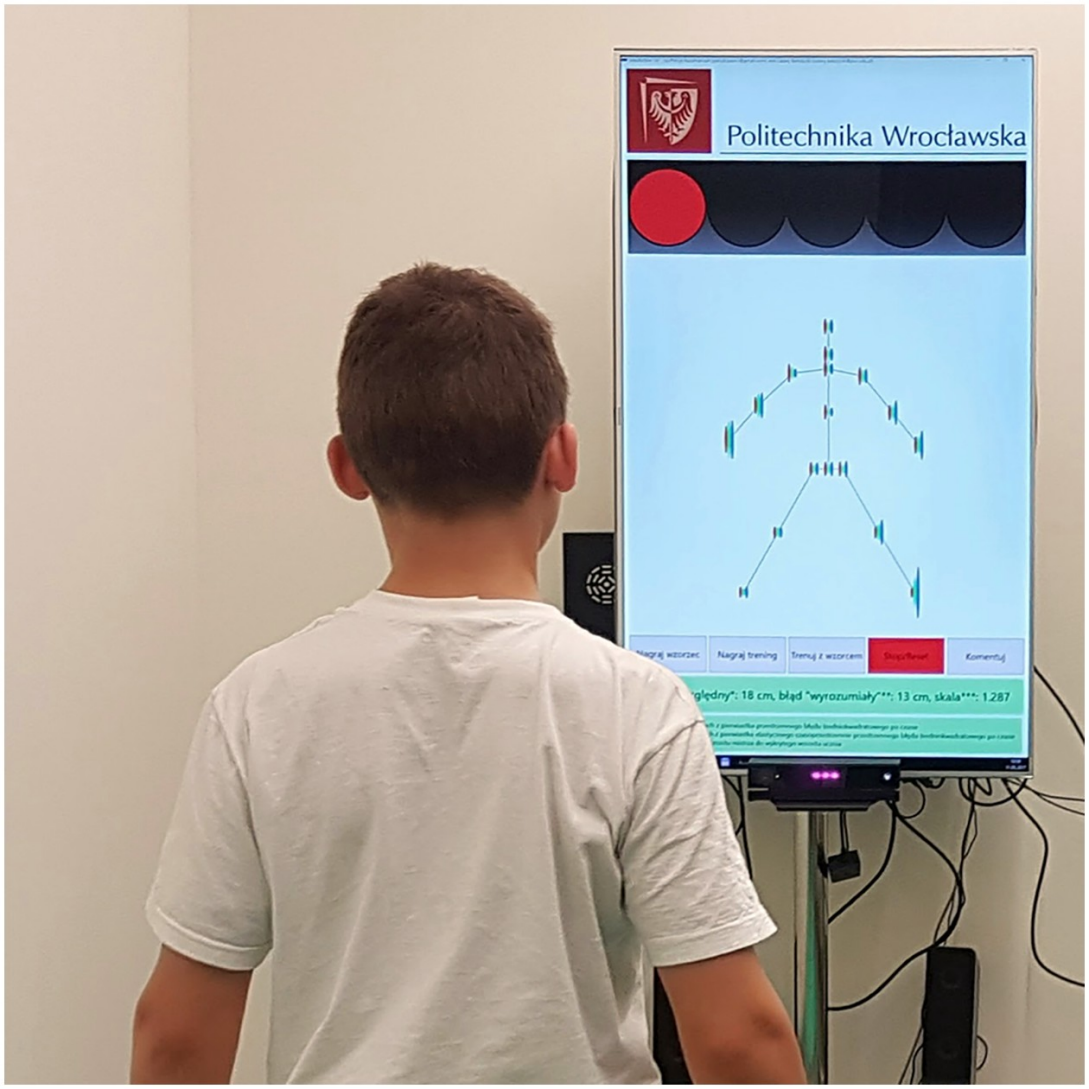
A young athlete training with the Kinect-based system. Graphical information displayed on the screen provides feedback (see text). The parent of the young individual depicted in the figure has given written informed consent (as outlined in the PLOS consent form) to publish these case details.

The practising athletes, supposed to mimic the master when performing the three judo throws, were supported by the InterAction software as follows. Two virtual skeletons were displayed on a large screen (1.4-m diagonal) simultaneously: one representing the previously recorded master and another representing the real-time movements of the athlete (Fig 2). The athlete’s height was estimated by InterAction based on Kinect’s readouts. Since the same had been done for the master, the height of the athlete was scaled with respect to the master’s height. The athlete was scaled onto the master rather than the master onto the practitioner so that all measurements would eventually be embedded in a unified probabilistic space. The same scaling ratio was applied in the *x*-, *y*-, and *z*-direction. On the need of scaling in sports, see, e.g., [19, 20], and references therein.

**Fig 3:**
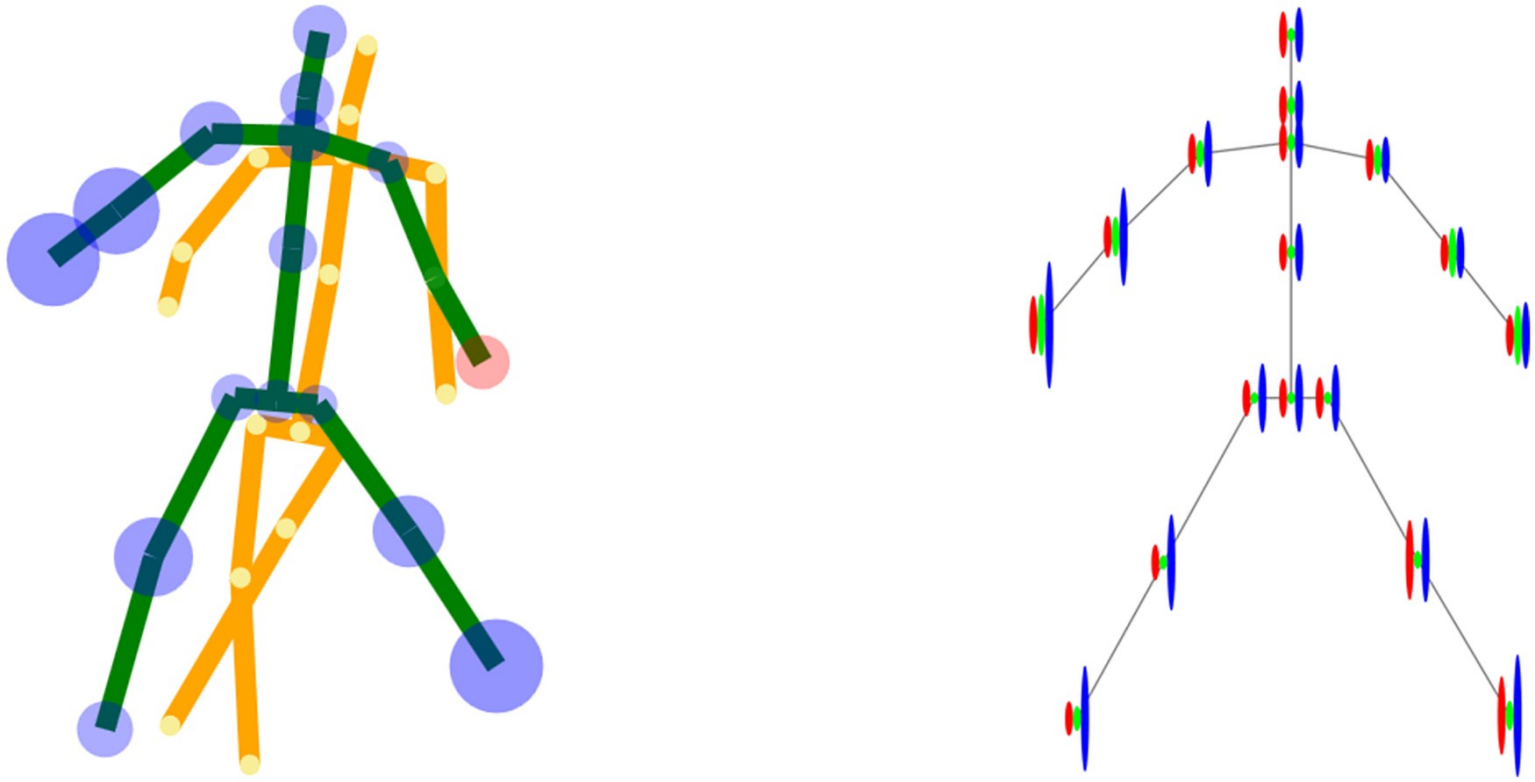
The real-time (left) and post-hoc (right) feedback presented visually to the athlete during Kinect aided training. In the real-time feedback (left), the master skeleton is displayed in yellow, whereas the skeleton of the practicing athlete is simultaneously displayed in green. The *z*-error (along the optical axis of the Kinect) of a joint position is colour coded in blue/red when the joint is located further/closer from Kinect than expected with respect to the master performance. In the post-hoc feedback (right), the size of the *x*-, *y*-, and *z*-error is graphically presented in red, green, and blue, respectively, for each of the joints.

Once the athlete had been scaled and vertically aligned to match the master on the screen, he was given a few seconds to match his posture and position to those of the master. Insofar as this is trivial in the *x*- and *y*-direction, as they are directly perceivable on the screen because mutual misalignment of the two skeletons in the *x*- and *y*-direction is easily noticeable, it is very difficult in the *z*-direction. That is why the athlete was supported in his real-time perception of the *z*-error with a dedicated encoding by means of colouring a badly positioned joint in either red or blue, depending on whether that joint was too close or too far from Kinect, respectively (Fig 3, left panel). In addition, the size of the joint representation on the screen was proportional to the absolute value of the *z*-error for that joint and was refreshed in real time, i.e. 30 times a second. The same encoding strategy was used during the actual performance.

A dedicated panel resembling traffic lights was used to help the athlete synchronise with the master in time as far as the beginning of the intended movement was concerned. The green light turned on when master’s movement occurred (Fig 2). Once the green light turned on the athlete had to start mimicking the master’s performance, given the feedback on the screen. At the end of a trial, the athlete was presented a skeleton scheme which graphically visualised his errors in the *x*-, *y*-, and *z*-direction for each individual joint (Fig 3, right panel). Based on this information, which was presented to him immediately at the end of a trial, he tried to improve in the next trial, which he initiated by performing a dedicated gesture recognized by Kinect.

In the evaluation of athletes’ performance, InterAction skips eight joints that Kinect fails to localise precisely. Those are: HandTipLeft, HandTipRight, ThumbLeft, ThumbRight, HandLeft, HandRight (but not wrists), FootLeft, and FootRight (but not ankles); see Fig 1, where they are marked as black. As derived from a one-minute recording (1,799 frames) with a still dummy as the subject, standard deviation of the localised spatial position for those joints exceeded 1 cm. Fortunately, these eight joints are located at the outermost ends of the limbs, which were not essential in our paradigm. The corresponding standard deviation for the remaining essential 17 joints (marked as white in Fig 1) was smaller than 1 cm in that recording.

### Data analysis

InterAction computes a scalar error for each joint of a performing athlete. This error is defined as the Euclidean distance between the spatio-temporal trajectory of a master’s joint in 3D and that of the corresponding joint of the athlete, divided by the square root of the number of recorded frames.

We evaluated athletes’ performance using this error measure in five comparison schemes: 1) Aided *vs* Controls in the 1st training session, 2) Aided *vs* Controls in the 5th training session, 3) Aided in the 1st *vs* 5th training session, 4) Controls in the 1st *vs* 5th training session, and 5) The difference of Aided in their 1st and 5th session *vs* the difference of Controls in their 1st and 5th session. Since a large percentage of these errors were not distributed normally as revealed by the Shapiro–Wilk test for normality [21] with *α* = 0.05, we used nonparametric tests in our analyses performed in MATLAB. For comparisons between the two groups (Aided *vs* Controls), the Wilcoxon rank sum test [22], equivalent to the Mann–Whitney *U* test [23], was used with *α* = 0.05. For comparisons between the two sessions (1st *vs* 5th), we used the Wilcoxon signed rank test for zero median [22] with *α* = 0.05. For each joint, each of the two compared samples consisted of 12 (the number of athletes) median errors computed across all 10 trials in a session.

If our data were normally distributed (and heteroscedastic) we could use the 2-way ANOVA to check interaction between the two factors, i.e., group and session. Unfortunately, examining interaction for data that are not normal is not easy. In his excellent review on nonparametric test of interaction [24], Sawilowsky showed that different nonparametric methods applied on the same dataset either rejected or failed to reject the null hypothesis about the presence of an interaction. Therefore, we abstained from elaborating on factor interactions, to avoid potentially false conclusions in this regard.

As argued in [24], the practice of using parametric tests when normality is not known based on their robustness to Type I error is the subject of much debate and a defence of this practice based on their robustness to Type II error is without merit.

Applying log-transformation to our samples reduced non-normality only partly, i.e., some, yet, not all, non-normal samples were turned into normal by the transformation. Apparently, apart from some samples being originally normal or lognormal, there were also some from more complex distributions. What is more, it is not known to which extent the log-transform would impact potential interaction.

Taking all the above into account we restricted ourselves to nonparametric statistics with the Wilcoxon signed rank and rank sum tests.

All data analysis routines are attached to this manuscript in the form of MATLAB scripts, which also draw Figs 4–11.

**Fig 4:**
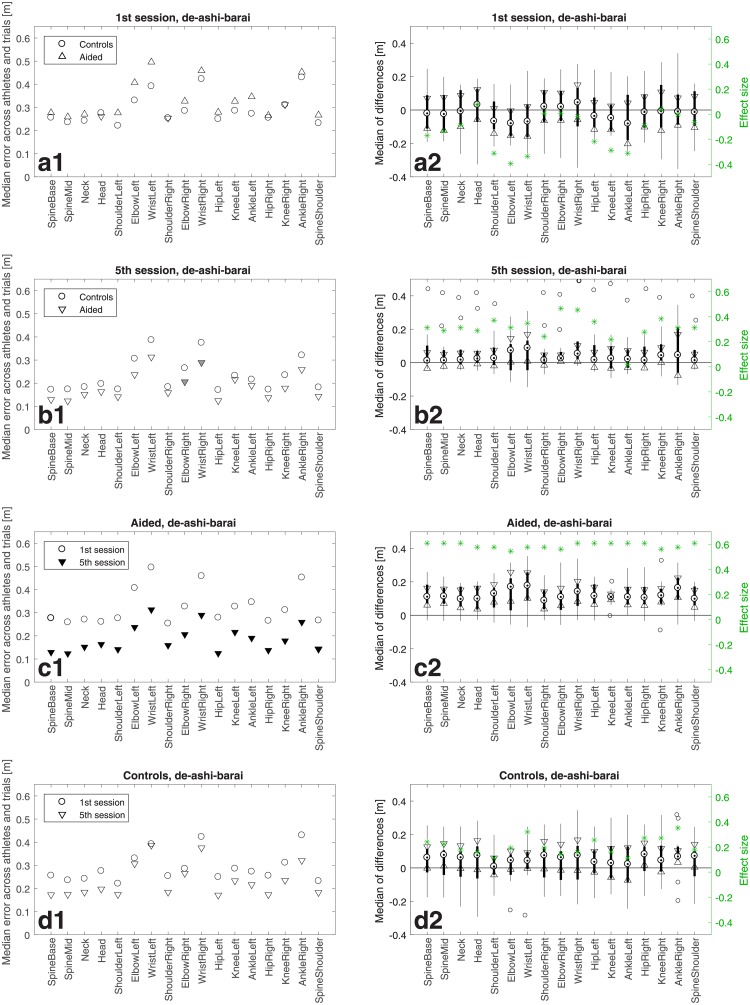
Results for *de-ashi-barai*. Panels in the left column show median error across athletes in a group and trials in a training session, in metres, with respect to the master performance for 17 body joints labelled along the horizontal axis. Consecutive rows show four types of comparisons: Aided *vs* Controls in the 1st and 5th session, the 1st *vs* 5th session for Aided and Controls; see legends. Solid triangles denote statistically significant differences with respect to the values represented by open circles, with grey and black triangles for *p* < 0.05 and *p* < 0.01, respectively. The right-column panels show the median of differences, marked with a black dot, with the 95% confidence interval bounded by the two triangles. The black bar limits correspond to the 25th and 75th percentiles, respectively. The whiskers extend to the most extreme data points not considered outliers, and the outliers are plotted individually as circles. The green stars denote the effect size defined as $z/\sqrt{2N}$, where *z* is the value of the *z*-statistic of the Wilcoxon rank sum test or the Wilcoxon signed rank test and *N* is the sample size.

**Fig 5:**
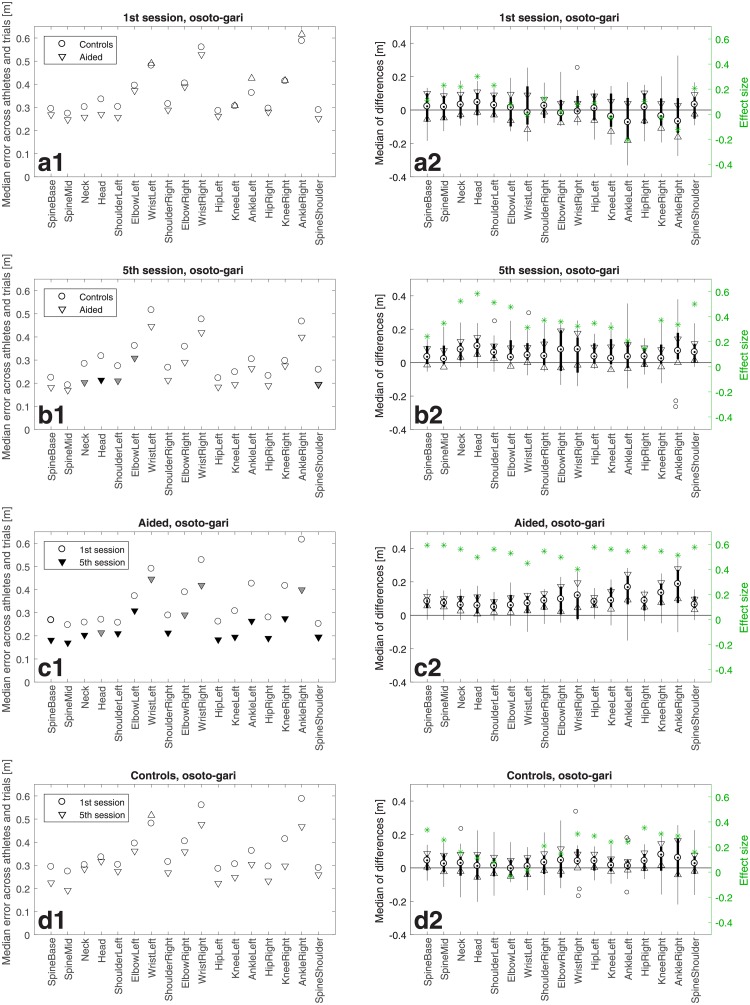
Like in Fig 4 but for *osoto-gari*.

**Fig 6:**
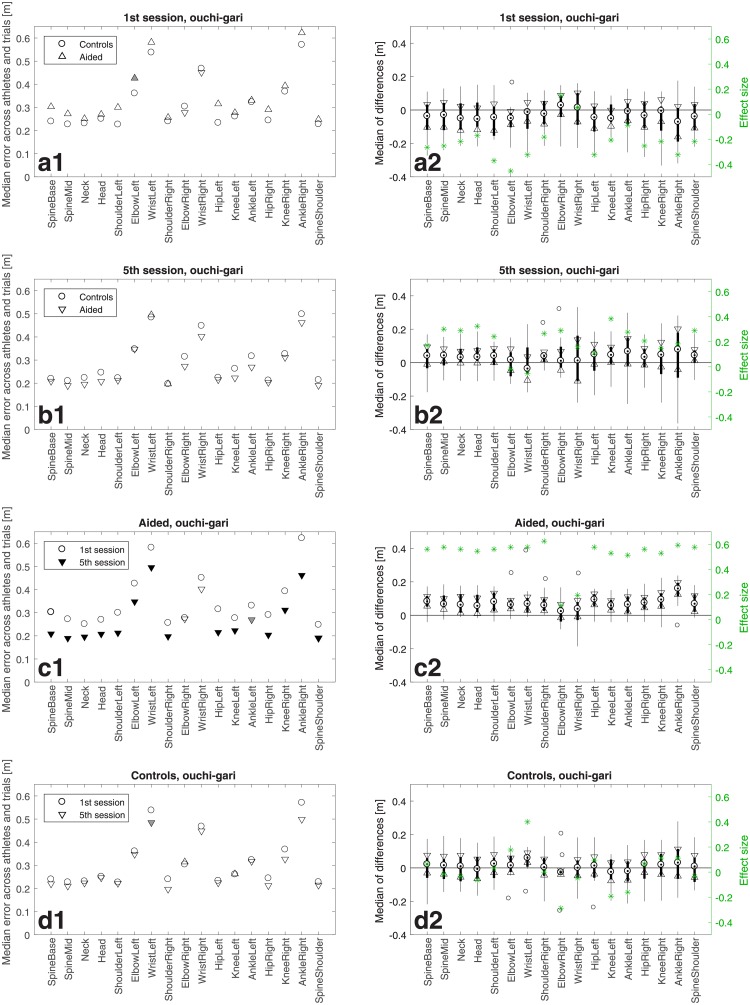
Like in Fig 4 but for *ouchi-gari*.

**Fig 7:**
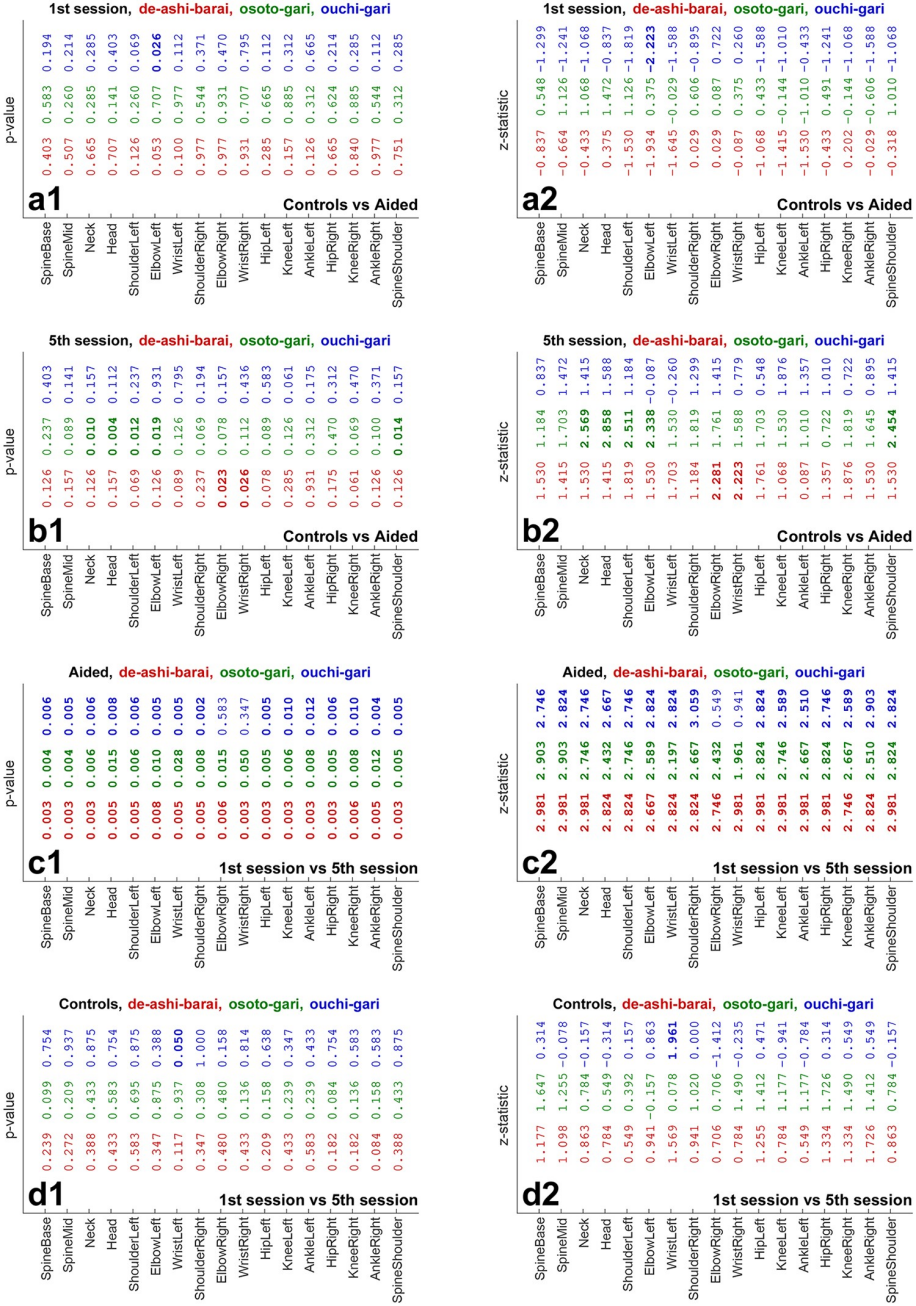
*p*-values (a1–d1) and *z*-statistics (a2–d2) for the comparisons depicted in Fig 4 (in red), Fig 5 (in green), and Fig 6 (in blue). Values are emphasized with bold font when *p* < 0.05.

**Fig 8:**
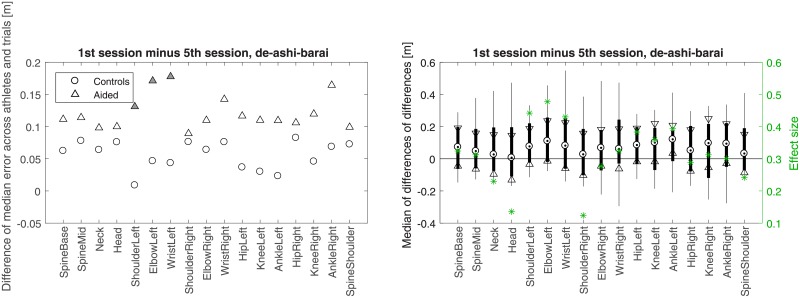
The difference of Controls’ performance in their 1st and 5th session *vs* the corresponding difference of Aided, for *de-ashi-barai*. For better readability, scales are different than those in Figs 4–6.

**Fig 9:**
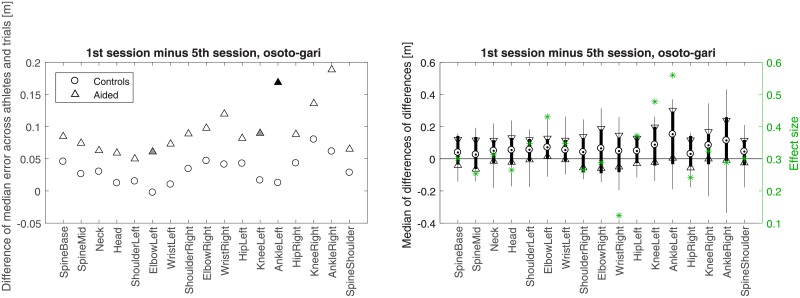
Like in Fig 8 but for *osoto-gari*.

**Fig 10:**
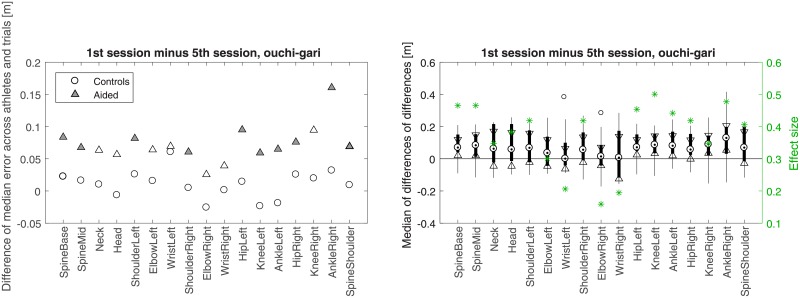
Like in Fig 8 but for *ouchi-gari*.

**Fig 11:**
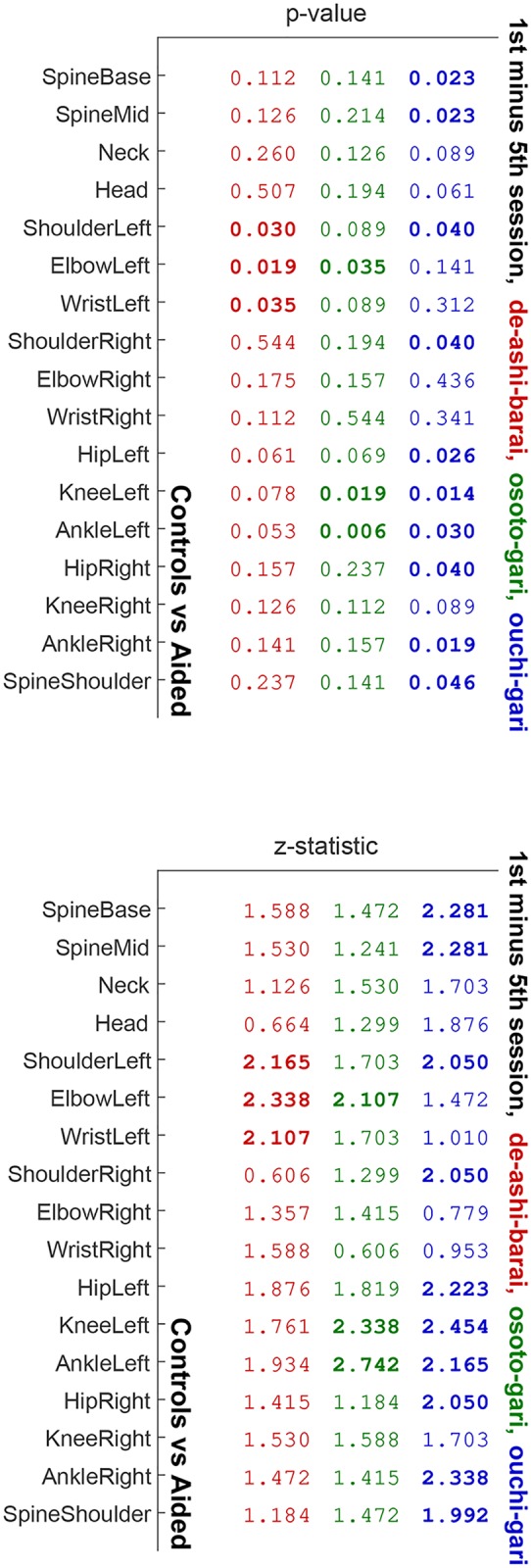
*p*-values (left) and *z*-statistics (right) for the comparisons depicted in Fig 8 (in red), Fig 9 (in green), and Fig 10 (in blue). Values are emphasized with bold font when *p* < 0.05.

## Results

Figs 4–6 show athletes’ performance for the three judo throws—*de-ashi-barai*, *osoto-gari*, and *ouchi-gari*, respectively—in the framework of the above described comparisons. In the four left-column panels of each figure, the symbols depict median errors across the 12 athletes in a group and 10 trials in a training session with respect to the master performance, for 17 individually considered body joints named with the labels placed along the horizontal axis. When a triangle symbol (pointing upwards/downwards for an increase/decrease of the error) is filled solid it means that the change with respect to the group/session represented by the open circle was statistically significant with *p* < 0.05 for grey triangles and *p* < 0.01 for black triangles.

The right-column panels show confidence intervals and effect sizes, which address the practical rather than purely statistical significance. The median of differences is shown for each joint as a black dot, whereas the 95% confidence interval is bounded by the two triangles. The black bar limits correspond to the 25th and 75th percentiles, respectively. The whiskers extend to the most extreme data points not considered outliers, and the outliers are plotted individually as circles. Note for panels c2 that with the exception of only two joints for *ouchi-gari* the confidence intervals do not contain zeroes, which confirms statistical significance of the results from Figs 4c1, 5c1 and 6c1. The green stars denote the effect size defined as $z/\sqrt{2N}$ [25, 26], where *z* is the value of the *z*-statistic of the Wilcoxon signed rank test or the Wilcoxon signed rank test and *N* is the sample size, i.e. 12 in this study.

In addition, Fig 7 shows *p*-values and *z*-statistics of all the comparisons from Figs 4–6, colour-coded to distinguish between the throws, i.e., in red for *de-ashi-barai*, green for *osoto-gari*, and blue for *ouchi-gari*.

Importantly, the top rows (panels a) of Figs 4–7 reveal no significant differences between the two groups (except for just a single joint for *ouchi-gari*) in the 1st session. This means that the two groups were statistically indistinguishable and hence fully comparable in terms of their abilities of mimicking the master performance at the beginning of the study [27]. This constitutes a good basis for further evaluation, because athletes’ initial skills should hence not bias the results of subsequent comparisons.

The most pronounced difference in performance was found in the Aided group between the 1st and the 5th session (3rd row in each figure, panels c). For *de-ashi-barai* (Fig 4) and *osoto-gari* (Fig 5), the improvement is clear and statistically significant for every single joint. For *ouchi-gari*, only two joints do not reveal statistical significance of the difference.

As for the second-row panels (b), i.e. the comparison of Aided *vs* Controls in the 5th training session, differences are less prominent, attaining statistical significance less often than in the case of comparisons between sessions for the Aided group. However, it is noticeable that the Aided group performs better, achieving smaller errors than Controls. It is also worth emphasising that for *ouchi-gari* (Fig 6b), improvement in the Aided group is clear given that this group performed somewhat worse (although not significantly worse) than the other group in the 1st session (Fig 6a). Athletes reduced their error more than Controls did, and that was the case for every throw. It is also important to note that in this Aided *vs* Controls comparison for the 5th training session, the Aided group performs significantly better than the Controls for two joints (ElbowRight, WristRight) in *de-ashi-barai* and for five joints (Neck, Head, ShoulderLeft, ElbowLeft, SpineShoulder) in *osoto-gari*.

A different picture emerges from the comparisons between the 1st *vs* 5th session for Controls. Athletes in this group made some, yet, rather minor progress for *de-ashi-barai* (Fig 4d) and *osoto-gari* (Fig 5d), and, in clear contrast to the Aided group (panels d), it was not statistically significant.

Figs 8–10 present another way of looking at the data. In each of the three figures, a comparison between the difference of Controls in their 1st and 5th session and the corresponding difference of Aided is shown. Such a differential approach provides additional insight regarding the improvement of the aided group with respect to the control group on a joint by joint basis (Wilcoxon rank sum test with *α* = 0.05), beyond what was presented indirectly in panels c and d of Figs 4–6. In Fig 10, significant improvement for most joints is seen in *ouchi-gari*, which was the most difficult throw for the athletes, as is further discussed in Discussion. As for the other two throws (Figs 8 and 10), the aided group improved noticeably, even if not statistically significantly, better than the control group.

Note that in contrast to Figs 4–6, the larger values for Aided than for Controls in Figs 8–10 stem from the differential nature of the depicted measure. Fig 11 provides all *p*-values and *z*-scores for these new comparisons.

## Discussion

In this study, which—as far as we are aware—is the first one to incorporate Microsoft Kinect in judo in a motor learning paradigm, we aimed at enhancing training efficiency and thus athletes’ performance. For each of the two groups of athletes, that is, Aided and Controls, the null hypothesis, which we hoped would be rejected in statistical evaluation, was that the level of performance would be the same in the last *vs* the 1st training session. For the aided group, this null hypothesis was rejected with *p* < 0.01 for all body joints in *de-ashi-barai*, for most of the joints in *osoto-gari* (with *p* < 0.05 for the remaining joints), and for most of the joints in *ouchi-gari* (with the exception of one joint with *p* < 0.05 and only two joints with *p* > 0.05). In contrast, there were no reasons to reject the null hypothesis in the control group in either of the three throws, with the exception of just one joint in *ouchi-gari* with *p* < 0.05.

Therefore, using Kinect clearly helped the athletes of the aided group improve their accuracy in mimicking master performances significantly in as few as five training sessions. In contrast, the control group, whose athletes were supported solely by a judo coach and mere video recordings of the same master performances but were restricted to train with InterAction and Kinect only in the 1st and 5th session—which was necessary for the purposes of a comparative study, as InterAction measures athletes’ performance using Kinect readouts—did not manage to improve as much as the aided group.

The lack of widespread significant differences between the two groups in their performance during the 5th training session can be explained in two ways. First, each of the two groups improved, although the control group improved less. Second, there were only five training sessions. The observed differences between the two groups would have likely been larger had the athletes of both groups participated in, say, 10 or 20 training sessions. However, they were limited by the time they could devote to the experiment.

The most problematic technique for the athletes turned out to be *ouchi-gari*. This seems to be rooted in a somewhat complex pattern of spatio-temporal trajectories of hand movements in the reference performance of the master, which was particularly difficult for the athletes to mimic. In addition, they had problems with coordinating those complex hand movements with the rest of their body in this technique. Fig 10 shows that using Kinect was particularly helpful for this throw, as indicated by statistically significant improvement for most of the joints.

As for a practical evaluation of the effectiveness of the Kinect aided training, the practically important difference would need to be defined as the smallest change, or threshold, which, when exceeded, would render using Kinect effective in real life. In professional sports, essentially any, even minor, improvement can be considered priceless. At a top level, it is often those small differences that distinguish an athlete as the winner of a competition. For example, differences of the order of centimetres can be decisive for a successful performance of a judo throw in real combat (and we found the Kinect able to provide sufficient precision in this regard for the essential joints; see Methods). This concerns aspects like the optimal trajectory of the limb action or the optimal body balance, especially given that the positioning of one body joint influences those of other joints. It is undoubtedly easier to correct these qualities in children, as they are free from habits stemming from years of training. But it is also vital to correct wrong habits in experienced athletes, where re-training of neural networks must occur so that the patterns of the athlete may coincide with the patterns of a top master. Of course, the more demanding a technique the more time it will take to improve and this is exactly where interactive, Kinect aided training can be of great help. In our study, the Kinect aided athletes managed to improve by as much as 10 cm or more within as few as five training sessions.

The virtual representation in the form of a skeleton with characteristic joints is similar to point-light models used in observational learning [13]. From the perception perspective, removing non-essential information can potentially facilitate skill acquisition, in comparison to, e.g., plain videos, although in [28, 29] there were no differences in results between the two modalities. Nonetheless, the critical asset in our paradigm was that the aided group combined both observational and physical practice via real-time visual feedback, which is advantageous [30, 31].

Some paradigms define the control group as one that does not have access to any reference material (e.g., [32]). That was not the case in our study, where the control group had access to video recordings of the master. The difference with respect to the aided group was that the latter was aided by Kinect with real-time and post-hoc feedback information on the errors, which was very helpful for the athletes. Indeed, when questioned, most athletes reported that they would like to see the Kinect system incorporated as permanent equipment in their judo clubs. We are convinced that this potential can be taken advantage of in other sports.

### Limitations and future directions

A methodological limitation of our study is the fact that the athletes were evaluated by the same setup that they trained with, which can introduce a bias. Optimally, another independent system could be used as an external reference. We were not in possession of such an additional system though. Another constraint is that only male athletes were investigated, although we have no reason to assume that female judo practitioners would learn differently to their male counterparts when using the present paradigm. In any case, we did not have access to a sufficient number of female athletes.

We did not perform a delayed retention test, although even if there had been one it would still be difficult to foresee to what extent the observed improvement would transfer to a better performance outside of the considered setup, for example, during real combat competitions. Even though a transference of skills enhanced by virtual reality setups to real life has been found in [33–35] in patients with Parkinson’s disease (see also an extensive review [36] on sensorimotor training in neurorehabilitation), real sports tournaments are characterized by many confounding variables which render those competitions somewhat complex in serving the potential role of objective evaluators as to whether or not a transfer of skills has taken place. Therefore, it would be rather difficult (at least in a short-time perspective) to correlate the results of the athletes of the two groups considered in the current study with their real-world performance.

It may seem intuitive that the athletes of the aided group had a potentially better chance of performing better in the 5th session compared to the athletes of the control group. But since the aim was to make the athletes perform closer to what the master is capable of, we can conclude that the proposed setup served its role very well. We believe that a long, regular practice with the proposed system, well beyond the time limitations of the current study, would eventually provide some, even if indirect, evidence of the aforementioned desired transference of the acquired or enhanced skills to real-combat conditions.

A technical limitation, already signalled in the Methods section, is the inability of Kinect to trace those joints that are not visible to its camera. A way of overcoming this limitation for complex movements could involve multiple Kinects. However, in contrast to the first generation, the second-generation Kinect cannot be controlled by a single computer in a multi-sensor setup. One could therefore use a few acquisition computers synchronized to the same external time server and match their readouts in post-processing, given that recorded frames can be time stamped with millisecond precision.

## Conclusion

We have presented results of a study aimed at an evaluation of the second-generation Microsoft Kinect as an aid to improve the performance of young judo athletes. We were particularly interested if using the Kinect can support motor learning with a virtual master performance serving as a role-model reference. Our results show that Kinect, when matched with specialised software, can be a useful means to supporting motor learning and therefore enhancing training efficiency, which translates to improved performance.

Last but not least, Kinect’s low cost, when compared to, e.g., gold-standard wearable electromagnetic sensors or marker-based optical movement capture systems, makes it an accessible and potentially popular tool for numerous virtual- and augmented-reality applications.

## Supporting information

S1 File Data. A.zip file containing:
A MATLAB .mat file with the data (athletes’ errors with respect to the master, as given by InterAction) that were subject to the MATLAB analyses whose results are presented in Figs 4–11. The .mat file contains two structures, aided and controls, each organized as a matrix of 12 athletes × 3 judo throws. Each entry of a 12 × 3 matrix is a structure containing sessions. Each session contains ten trials and each trial consists of the errors for the 17 considered body joints. See Methods for a detailed description.MATLAB .m scripts that perform the aforementioned analyses and plot Figs 4–11.
(ZIP)
